# Evaluation Index System of Economic and Social Development Pilot Area Based on Spatial Network Structure Analysis

**DOI:** 10.1155/2022/3019440

**Published:** 2022-05-09

**Authors:** Jing Tu

**Affiliations:** School of Economics, Xihua University, Chengdu 610039, China

## Abstract

In order to improve the evaluation effect of the economic and social development pioneer area, this paper constructs the evaluation index system of the economic and social development pioneer area based on the spatial network structure analysis method and obtains an intelligent analysis system. Moreover, from the perspective of economic development information flow, this paper uses information flow direction analysis method and advantage flow analysis method to discuss the structural characteristics of urban economic development network in the economic belt, providing new methods and perspectives for the study of urban economic development flow. In addition, this paper attempts to propose a universal method for quantitative research on the “flow space” structure of urban economic development as the forward-looking content of urban economic development. According to the simulation test results, it can be seen that the evaluation index system of the economic and social development pilot area based on the analysis of the spatial network structure proposed in this paper has a good effect.

## 1. Introduction

The new development concept is an inseparable organic whole. In this organic whole, innovation is the internal driving force leading development and must be placed at the core of development, including innovation in theory, system, technology, culture, and other aspects. Coordination is the principal requirement of development, and the concept of coordinated development pays more attention to integrity, systematization, and balance and must correctly handle major relations in development and solve the problem of unbalanced and insufficient development [1]. At the same time, green is a necessary condition for development, and green development focuses on solving the problem of harmonious coexistence and coexistence between man and nature. While continuing and emphasizing adherence to the basic national policy of resource conservation and environmental protection, it is necessary to achieve synergy between economic and social development and ecological and environmental protection and create a good production and living environment for the people. Opening up is the environmental layout of development. To solve the problem of developing internal and external linkages, a higher-level open economy must be developed to expand opening up and promote reform and development [2]. In addition, sharing is the fundamental purpose of development, focusing on solving social fairness and justice issues and continuously promoting the common prosperity of all people. The five development concepts are interconnected, and promote each other, constituting a brand-new concept and action guide to guide and lead today's development. Moreover, high-quality development under the guidance of new development concepts will surely become the normal state of development in the new era [3].

At present, the research on the airport economy at home and abroad is generally summarized from the aspects of case analysis and planning and construction, focusing on basic theoretical research such as basic concepts, characteristics, and development models at the macro level. However, there is a lack of specialized research on the airport economy industry from the perspective of regional economics, management, and industrial economics, as well as microsubjects. It is necessary to select and plan the airport industry based on the development time sequence by using the analytic hierarchy process in combination with the types of airport economic industries. At the same time, it is necessary to rationally plan and lay out the industrial space according to the industrial space structure layout of the airport area and combine the regional characteristics of the economic demonstration area so as to enrich the theoretical research system of the airport economy.

Based on the spatial network structure analysis method, this paper constructs the evaluation index system of the economic and social development pioneer area and obtains an intelligent analysis system, which provides a reference for the subsequent evaluation of the economic and social development pioneer area.

## 2. Related Work

The evaluation theory of economic and social development is a process of continuous evolution, and the evolution process is consistent with the development of the context and the theory of economic and social development. Through the study of relevant literature, it can be seen that the theory of economic and social development will affect the theory and connotation of measurement and evaluation. The changes of economic and social development theory with time and environment will inevitably lead to changes in the content of economic and social measurement and evaluation [4].

The social development concept of one-sided pursuit of economic growth has its profound and complicated historical reasons. The two world wars severely damaged the economy of the traditional capitalist powers. With the collapse of the world colonial system, the rise of independent democratic movements in the colonies directly shook the economic foundation of imperialism [5]. After the Second World War, the primary task of traditional capitalist powers was to restore and develop their own economies. Countries that have just gotten rid of the colonial system and become independent and countries that have just begun to industrialize have become developing countries. The primary task of developing countries is to develop their economies. In this way, the world is pursuing economic growth. Therefore, the development of this period mainly refers to the economic development. Due to special historical reasons, people at that time believed that the cause of social problems was the low economic aggregate, and rich material wealth could solve all social problems. Typical theories include Lewis's [6] dual structure theory, Rostow's economic development stage theory [7], Kuznets' statistical theory [8], and Chenery's econometric theory [9]. Under the guidance of this concept, the total economic volume has increased significantly, the progress of other aspects of society has been slow, or even counterproductive, and problems such as social unrest, political corruption, and unfair income distribution have emerged one after another. The concept of one-sided pursuit of economic growth began to be questioned.

Under the guidance of the one-sided pursuit of economic growth, other aspects of society have not achieved the expected progress, and people have begun to realize that simple economic growth cannot solve problems such as poverty, unemployment, and inequitable distribution [10]. The development theory has evolved from economics to a multidisciplinary comprehensive theory, which affirms the basic status of economic growth while taking into account other aspects of society. However, the focus of this period was mainly on social welfare, aiming to solve poverty, unemployment, and inequitable distribution. The focus is on raising the income of the poor, increasing the employment rate and improving the social security system [11]. Literature [12] believes that development should include economic, political, and cultural development and believes that as long as poverty, unemployment, and inequality are reduced, it belongs to development. Literature [13] proposed that development is a multidimensional process that includes the combination of the entire economy, society, culture, technology, ecology, and other elements and internal reorganization, including changes in economic aggregates, economic income growth, social systems, social structures, and even people's beliefs, habits, and other related content that cannot be regarded as a simple process.

In the process of industrialization, human beings have experienced an extensive economic development model. In this process, human beings exploit resources plunder, destroy the ecological environment, pursue short-term interests, and make economic development unsustainable [14]. Literature [15] believes that sustainable development mainly includes two cores, namely, the welfare problem in the economic growth period and the constraint problem of economic growth. The optimal growth path under different intergenerational goals and resource constraints is studied, and it is found that the optimal growth path is the path of sustainable development most of the time. Some scholars put forward another view. Literature [16] believes that the systematic interpretation of the connotation of “sustainable development” should be carried out from three aspects: economic sustainability, environmental sustainability, and social sustainability. With the deepening of research, the academic community has further enriched the theoretical framework of sustainable development and believes that the theoretical connotation of sustainable development includes five aspects: multidimensional development, common development, fair development, coordinated development, and efficient development. The indicator system of the United Nations Commission on Sustainable Development covers the four major systems of economy, society, environment, and system, including 142 indicators designed by driving force, state, and response model. The US Sustainable Development Indicator System consists of ten goals, including 54 indicators including health and the environment, equality, sustainable society, economic prosperity, public participation, protection of nature, and education. The UK sustainable development indicator system includes 21 aspects including economy, transportation, overseas trade, energy, land use, water resources, forests, atmosphere, waste, and radioactivity, with a total of 123 indicators [17].

## 3. Analysis Model of Spatial Network Structure

The city network mainly refers to the connection between cities. The relational data between cities is particularly critical to the study of urban networks, which can be roughly divided into attribute methods and relational methods. The data with realistic or abstract meanings that can reflect and carry various associations between cities, such as the flow of people, material exchange, and information exchange between cities, can be understood as relational data. The traditional urban network research is mostly carried out from the mode of urban hierarchy, scale, core-periphery, and so on. However, the urban network studied in this paper refers to the urban network in which the economic development information flow represents the abstract meaning perspective, and this paper reveals the connection between urban tourism through the abstract research perspective.

The central place refers to the place where goods and services can be provided to consumers in the surrounding area, such as a residential distribution point, a commercial center, or a city. Centrality is the degree to which a central place plays a central role relative to its surrounding areas, that is, its relative importance. Centrality can be expressed as follows [18]:

(1) $$\begin{array}{c} C=C_{1}-C_{2}. \end{array}$$

Among them, *C*_1_ is the total amount of goods and services provided by the center, *C*_2_ is the amount of goods and services provided by the center for itself, and *C* is the centrality, which refers to the amount of goods and services provided by the center to surrounding areas.

The central place theory is regarded as the basic theory of urban geography and regional economics research, as well as the foundational theory of urban system spatial structure research. The central place theory is a theory about the size of urban functions and the distribution of spatial structure in the region. It reveals the basic characteristics of the spatial structure of the central place system. In the tourism practice research, the urban tourism architecture mainly studies the system level division, the spatial layout in a certain area, and the connection between each other. The urban tourism grade is analyzed to provide support for the next regional tourism spatial structure.

The core idea of growth pole theory is that economic growth does not occur at the same speed in every location at the same time. Generally, the growth rate of a dominant economic sector or an innovative industry is the fastest, and these sectors and industries tend to gather in the best location. The location is usually a large- or medium-sized city within the region. Therefore, these large- and medium-sized cities gradually form regional growth poles and drive regional development through diffusion effects.

This theory provides a basis for the priority development of regional tourism. Due to the comprehensive characteristics of the tourism industry, the linkage effect of the tourism industry is extended to a larger area through the gathering and diffusion of regional tourism growth poles. In addition, regional tourism is not initially developed in a balanced way. According to the development model of this theory, priority development areas can be used to drive tourism development in other regions so that it can play a role in the aggregation and diffusion of growth points and drive the economic growth of the entire regional tourism industry. The theory is mainly used in the two forms of the point axis structure and the network structure in the tourism space structure.

The point-axis theory is formed on the basis of the growth pole theory, which holds that the “point” is the node (city) at all levels, and the “axis” is the industrial belt formed by connecting the nodes at all levels in a certain direction. The point-axis structure is formed by a number of isolated nodes through the connection of the development axis and gradually develops to form a certain spatial network structure. Cities in a certain area are distributed hierarchically, so the development axis connecting each city is also hierarchical, and cities and axes with different levels have different attractiveness.

In the related research, the measurement methods used by most scholars to study the evolution law and trend of regional tourism development differences are basically based on the relevant methods of income distribution differences in economics. The specific measurement methods used in this paper are as follows:

(1) *Standard Deviation (VOC)*. It is the arithmetic square root of the ratio between the sum of squared deviations of the sample data and the total sample size. It reflects the absolute degree of dispersion between sample individuals and can be used to represent the absolute difference in economic scale between cities. The larger the value, the greater the absolute difference.
  (2) $$\begin{array}{c} \text{VOC}=\sqrt{\frac{\sum_{i=1}^{n}{\left(x_{i}-\bar{x}\right)}^{2}}{n}}. \end{array}$$
(2) *Coefficient of Variation (CV)*. It is the quotient between the standard deviation and the mean. It can eliminate the interference of different sample averages on the degree of sample variation, reflect the relative dispersion of samples, and can be used to characterize the relative difference of tourism scale between cities. The smaller the value, the smaller the relative difference.
  (3) $$\begin{array}{c} \text{CV}=\sqrt{\frac{\sum_{i=1}^{n}{\left(x_{i}-\bar{x}\right)}^{2}/n}{\bar{x}}}. \end{array}$$
(3) *Gini Coefficient*(*G*). It is combined with the coefficient of variation to determine the relative difference of urban tourism scale. The larger the value is, the larger the tourism scale gap between cities is.
  (4) $$\begin{array}{c} G=1+\frac{1}{n}-\frac{1}{n^{2}\bar{y}}\left(y_{1}+2y_{2}+3y_{3}+\cdots +ny_{n}\right). \end{array}$$
(4) *Growth Rate*. It can analyze the differences in the speed of regional tourism development, but the bases of cities are different, and the same growth rate will lead to large differences in actual growth. In order to eliminate the above situation, this paper introduces the relative development rate (Nich) to measure the spatial difference of the relative development speed of urban tourism in the economic belt.
Nich is the relationship between the change of the economic development scale of each city in a certain period and the change of the average economic development scale of the whole region in the same period:
  (5) $$\begin{array}{c} \text{Nich}=\frac{\left(Y_{2i}-Y_{1i}\right)}{Y_{1}-Y_{2}}. \end{array}$$
In the formula, *Y*_1*i*_,*Y*_2*i*_ is the economic development scale of city *i* at time 1 and 2, and *Y*_1_,*Y*_2_ is the average economic development scale of the region at time 1 and 2. Nich > 1 means that the growth rate of the economic development scale of city *i* is higher than the regional average level, 0 < Nich < 1 means that the growth rate of the economic development scale of city *i* is lower than the regional average level, and Nich < 0 means that the economic development scale of city *i* has negative growth.
(5) *Herfindahl Coefficient*(*Hn*). It is an index reflecting the scale and agglomeration degree of urban economic development. The larger the value, the higher the degree of concentration, and the more uneven the development.
  (6) $$\begin{array}{c} H_{n}=\sum_{i=1}^{n}p_{i}^{2}. \end{array}$$
(6) *First Degree (S)*. It is the quotient between the measures of the economic development scale of the first and second largest cities. It reflects the aggregation degree of urban economic development scale distribution. The larger the value, the more concentrated the distribution. When *S* > 2, the regional structure is considered unreasonable, and it is called the primacy distribution. When *S* < 2, the regional structure distribution is considered to be relatively reasonable.
  (7) $$\begin{array}{c} S=\frac{P_{1}}{P_{2}}. \end{array}$$

In formulae (1)–(6), *X*_*i*_ is the value of index *i*, *Y*_1_, *Y*_2_, *Y*_3_ ⋯ *Y*_*n*_ is the index value from large to small, $\bar{X}$ and $\bar{Y}$ are the mean values of the indexes, and *n* is the total number of samples, *P*_*i*_ is the proportion of index *i*, and *P*_1_, *P*_2_ is the index value of the first and second largest cities.

### 3.1. Research Methods of Economic Development Scale Distribution

In order to better present the overall distribution of the economic development scale of cities in the economic belt. In this paper, the Rothko-type rank-scale distribution is used to measure the economic development scale distribution of cities in the economic belt. The formula is [19]

(8) $$\begin{array}{c} P=KR^{-q}. \end{array}$$

Among them, *P* is the scale of urban economic development, *R* is the rank, *K* is the ideal scale of the first city, and *q* is the concentration index. According to the *q*-value, the scale distribution of urban economic development is divided into first type (*q* ≥ 1.2), concentrated type (0.85 < *q* < 1.2), and balanced type (*q* ≤ 0.85).

In order to scientifically use the sample data for regression analysis, the natural logarithm of formula (7) is taken and sorted out:

(9) $$\begin{array}{c} \ln\;p=\ln\;k-q\;\ln\;R. \end{array}$$

This paper uses the index of the total number of people received by the city's economic development to measure its functional scale and the index of the proportion of total economic development revenue to GDP to measure its functional status. Moreover, this paper uses Nelson's method to define the functional strength of each city. First, the sample mean $\left(\bar{\text{X}}\right)$ of each city's functional indexes is calculated as the threshold for judging urban specialized departments; then, the sample standard deviation (*σ*) of each city's functional indexes is calculated, and then the coefficient (*K*_*i*_) of each city's functional intensity is calculated.

(10) $$\begin{array}{c} {\bar{X}}_{i}=\frac{1}{n}\sum_{i=1}^{n}X_{i},\\ \sigma_{i}=\sqrt{\frac{\sum_{i=1}^{n}{\left(X_{i}-\bar{X}\right)}^{2}}{n}},\\ K_{i}=\frac{X_{i}-\bar{X}}{\sigma }. \end{array}$$

Each dimension is divided into 3 intervals according to the value of *K*_*i*_. The specific description is as follows. In the dimension of functional scale, it is divided into small, medium, and large cities. In terms of functional status, cities are divided into low, medium, and high specialization cities.

The mean value of the total number of people received by the economic development of cities in the economic belt is expressed by $\bar{X}$, and the standard deviation is expressed by *σ*. After calculation, it is found that the original sample of functional scale is nonnormal. Therefore, this paper corrects it as follows:

(11) $$\begin{array}{c} T_{i}=\left\{\begin{array}{c} \text{small}-\text{scale }T_{i}\leq \bar{X}\tau ,\\ \text{medium}-\text{sized }\bar{X}\tau <T_{i}<\bar{X}\tau +\sigma_{i},\\ \text{large }T_{i}\geq \bar{X}\tau +\sigma_{i}, \end{array}\right.\\ S_{i}=\left\{\begin{array}{c} \text{Low specialization }S_{i}\leq {\bar{X}}_{S},\\ \text{Specialization in secondary schools }{\bar{X}}_{S}<S_{i}\leq {\bar{X}}_{S}+\sigma_{i},\\ \text{High specialization }S_{i}\geq {\bar{X}}_{S}+\sigma_{i}. \end{array}\right. \end{array}$$

Among them, *T*_*i*_ is the functional scale of urban economic development, and *S*_*i*_ is the functional status of urban economic development.

**Figure 1:**
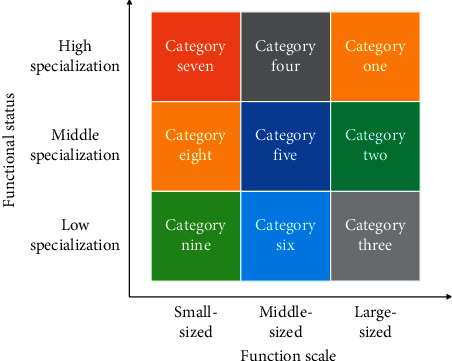
Types of urban economic development functions.

The two dimensions are combined with each other to form 9 types of urban economic development functions (Figure 1).

With the rapid development of information technology, the popularization of the Internet and the economic development reservation system has largely changed the market conditions of the economic development industry. Moreover, the application of information technology in the economic development industry is becoming more and more extensive, and the influence of information flow on the economic development flow is gradually increasing. The guidance of economic development information flow to economic development flow is a process of information decision-making and implementation; in this process, “quasitourists” have been largely controlled by economic development information flow. On the one hand, the flow of economic development information enhances the flow of economic development, and on the other hand, the two can even replace each other in quantitative relationship. In the context of the rapid development of the Internet, the purchasing decisions and behaviors of economic developers are increasingly dependent on the Internet. Website information flow is the basic form and the only medium of Internet information transmission, and it plays a huge guiding role in the generation and change of people flow and is the most influential “flow” among many “flow” elements. Therefore, from the perspective of economic development information flow, this paper avoids the obstacles caused by the lack of statistics on domestic economic development flow data.

From the perspective of economic development information flow, this paper uses information flow direction analysis method and advantage flow analysis method to discuss the structural characteristics of urban economic development network in the economic belt, providing new methods and perspectives for the study of urban economic development flow. Moreover, this paper attempts to propose a universal method for quantitative research on the “flow space” structure of urban economic development as a forward-looking content of urban economic development so as to make up for the deficiencies of related research caused by the lack of information on the flow and flow of domestic actual economic development and to provide certain basic materials for later scholars to study the flow of economic development information. To a certain extent, it provides decision-making reference for economic development marketing, economic development e-commerce and economic development planning and provides a scientific basis for promoting the integrated construction and development of economic development.

This paper introduces *C*-value and *D*-value as two indicators to measure the status and level of each city in the urban economic development network. The formula is

(12) $$\begin{array}{c} C=\text{Ln}\frac{C_{c}}{C_{s}},\\ D=C_{c}-C_{s}. \end{array}$$

Among them, *C*_*c*_ represents the amount of economic development information output by a city, and *C*_*S*_ represents the amount of economic development information received by the city. If a city's economic development information output is greater than its information reception, that is, *D* > 0, the city is the control node (city) of the economic development information flow network. On the contrary, the city is considered as a subsidiary node (city).

In the process of data processing, it is found that, under normal circumstances, the amount of economic development information received by popular cities or cities with more developed economic development industries is often greater than the amount of economic development information output. The information flow of economic development from low to high is relatively large, while the amount of economic development information output from high to low is less. Therefore, *C*_*c*_ represents the amount of economic development information received by a city, and *C*_*S*_ represents the amount of economic development information output by the city.

The basic idea of the dominant flow analysis method is to judge the status of a city (A) in the urban system according to the maximum flow of economic development information of a city (A), the scale of urban economic development, and the flow between the city (A) and other cities. From this, the position of the city (A) in the network structure is obtained, and the position determines its influence in the spatial interaction. On this basis, Song Wei and other scholars divided cities into three types: dominant, subdominant, and subordinate cities. Based on the scale of urban economic development level obtained in the previous study, the level of the dominant stream is divided. The detailed contents are explained as follows. If the largest economic development information flow of city (A) is to city (B) with a smaller economic development scale, then city (A) is considered to be dominant. If city (B) has the largest economic development information flow to a city with a relatively large economic development scale, and at the same time there is one or more cities (*C*_1_, *C*_2_⋯) whose economic development scale is smaller than the largest economic development information flow to it (B), then city (B) is considered to have secondary dominance. If city (C) has the largest economic development information flow to the subdominant city (B), and there is no city (D) whose economic development scale is smaller than the largest economic development information flow to it (C), then it is determined that city (C) has a subordinate attribute.

## 4. Evaluation Index System of Economic and Social Development Pilot Area Based on Spatial Network Structure Analysis

**Figure 2:**
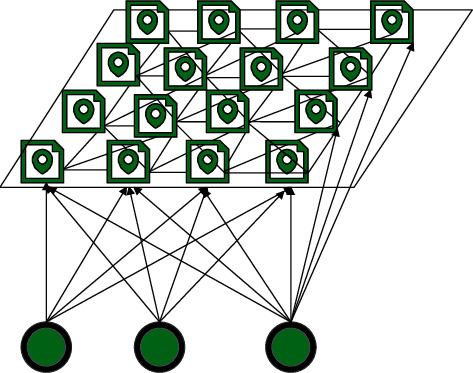
Kohonen network structure.

The Kohonen network simulates the structure of neurons in the cerebral cortex as a two-dimensional spatial lattice. Functionally, through the interaction and competition between neurons in the network, the clustering function and self-organization and self-learning functions of brain information processing are simulated. The Kohonen network structure is shown in Figure 2, which consists of an input layer and a competition layer.

**Figure 3:**
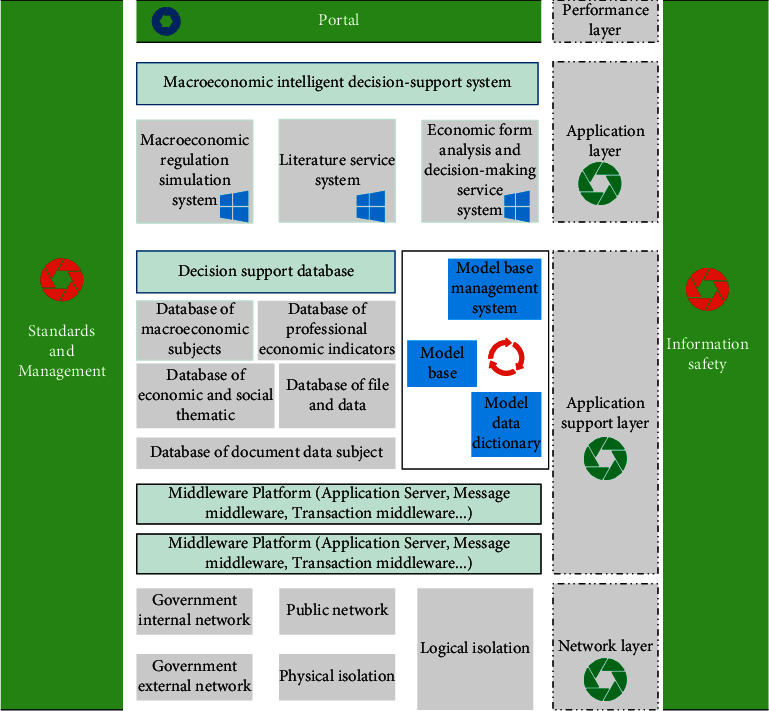
Overall architecture of the system.

This paper makes comprehensive use of multidisciplinary theories and technologies such as systems science, information science, dynamic econometrics, artificial intelligence, data warehouse, data mining, online analytical processing, and intelligent decision-making analysis tools and methods. Through the collection, storage, analysis, processing, and presentation of various data and related information, high-level information services and support are provided for leaders, and scientific and standardized decision-making is realized. The overall architecture of the system is shown in Figure 3.

**Figure 4:**
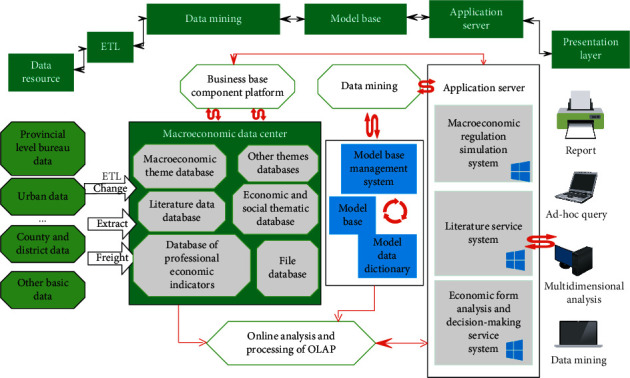
System flowchart.

The flowchart of the macroeconomic intelligent decision support system is shown in Figure 4.

**Figure 5:**
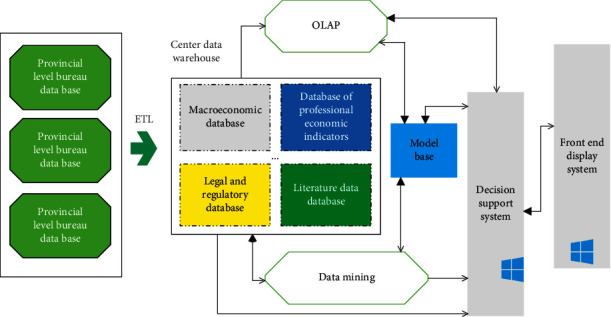
Schematic diagram of the decision support system based on data warehouse.

**Figure 6:**
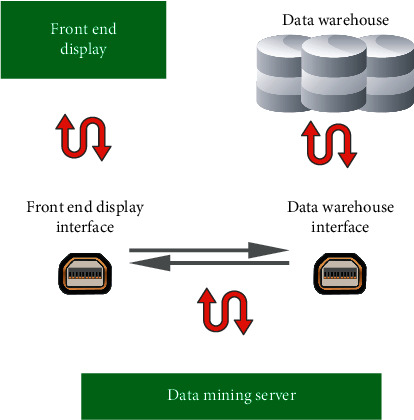
Interface design of data mining server.

The intelligent decision support system based on data warehouse takes data warehouse and model base (MB) as the main structure, supports online analytical processing, and applies data mining to knowledge discovery in the database, as shown in Figure 5:

The data mining server extracts data from the data warehouse and then sends the results to the front-end for display after mining and processing. The interface design of the data mining service is shown in Figure 6 below.

**Figure 7:**
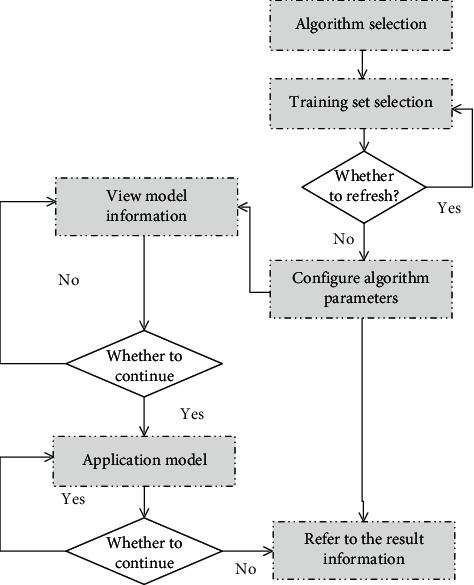
Overall activity diagram of data mining module.

The mining steps of the data mining system are as follows: (1) The system selects an algorithm. (2) The system selects the training set and determines whether to refresh. If it needs to be refreshed, the system repeats step 2; otherwise, it goes to step 3. (3) The system configures the algorithm parameters. (4) The system checks the model information and chooses to continue to step 5; otherwise, it goes back to step 3. (5) The system uses the model to determine whether to refresh. If it needs to be refreshed, the system repeats step 5; otherwise, it goes to step 6. (6) The system checks the result information. The overall activity diagram of the data mining module is as shown in Figure 7.

**Figure 8:**
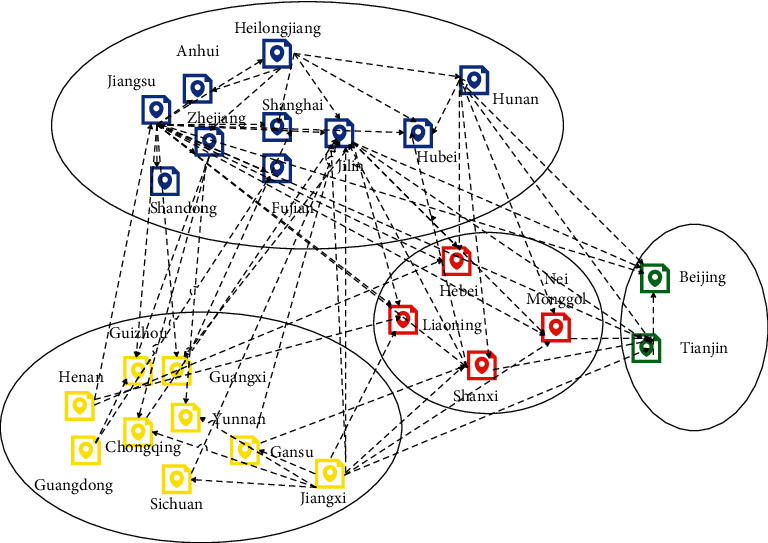
Analysis model of spatial network structure.

Figure 8 shows the analysis model of the spatial network structure constructed in this paper.

**Figure 9:**
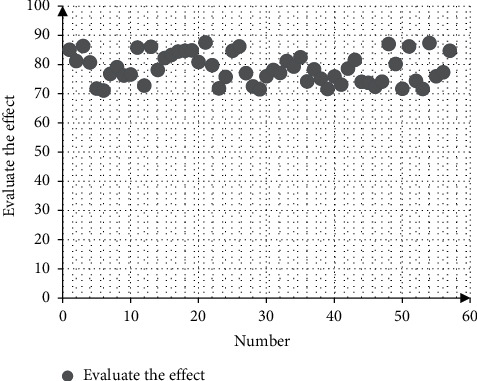
Statistical diagram of the evaluation of economic and social development pilot areas.

This paper obtains a large amount of data from the network to verify the effect of the model in this paper and calculates the effect of the model constructed in this paper in the evaluation index analysis of the economic and social development pilot area and compares it through multiple sets of data. Moreover, this paper obtains the results shown in Table 1 and Figure 9.

From the above research, it can be seen that the evaluation index system of the economic and social development pilot area based on the analysis of the spatial network structure proposed in this paper has a good effect.

## 5. Conclusion

The construction and development process of the “two first districts” is the implementation of the five development concepts of innovation, coordination, greenness, openness, and sharing and is the development under the strategic guidance of the five development concepts. Therefore, the construction of the evaluation index system of the economic and social development pilot zone must be based on five development concepts. Moreover, it needs to take the five development concepts as the first-level indicators for evaluation and further select the second-level indicators and the third-level indicators according to their connotations and the actual construction. In addition, it provides a theoretical reference for the formation of the basic framework and the selection of specific indicators for the evaluation index system of the economic and social development pilot zone. At the same time, it can reveal the status quo, characteristics, and problems of economic and social development under the guidance of new development concepts and has certain practical value for guiding government departments to solve economic and social development problems according to the development requirements of new concepts. Based on the analysis method of spatial network structure, this paper constructs the evaluation index system of the economic and social development pilot area and obtains an intelligent analysis system. The simulation test study shows that the evaluation index system of the economic and social development pilot area based on the analysis of the spatial network structure proposed in this paper has a good effect.

## Figures and Tables

**Table 1:** Evaluation of the evaluation effect of the economic and social development pilot area.

Number	Evaluate the effect	Number	Evaluate the effect	Number	Evaluate the effect
1	85.04	20	80.86	39	71.63
2	81.19	21	87.61	40	75.90
3	86.35	22	79.78	41	73.16
4	80.73	23	71.85	42	78.66
5	71.80	24	75.74	43	81.58
6	71.07	25	84.73	44	74.08
7	76.79	26	86.22	45	73.76
8	79.12	27	77.03	46	72.41
9	76.22	28	72.53	47	74.15
10	76.56	29	71.51	48	87.06
11	85.82	30	76.00	49	80.16
12	72.76	31	78.15	50	71.73
13	86.10	32	77.13	51	86.23
14	78.13	33	81.22	52	74.45
15	82.19	34	79.27	53	71.64
16	83.32	35	82.48	54	87.36
17	84.48	36	74.20	55	75.99
18	84.74	37	78.38	56	77.32
19	84.83	38	75.16	57	84.74

## Data Availability

The labeled dataset used to support the findings of this study are available from the corresponding author upon request.

## References

[1] Barde S. (2015). Back to the future: economic self-organisation and maximum entropy prediction. *Computational Economics*.

[2] Bhattacharya D., Mukhoti J., Konar A. (2019). Learning regularity in an economic time-series for structure prediction. *Applied Soft Computing*.

[3] Daksiya V., Su H. T., Chang Y. H., Lo E. Y. M (2017). Incorporating socio-economic effects and uncertain rainfall in flood mitigation decision using MCDA. *Natural Hazards*.

[4] Ferramosca A., Alejandro H., Limon D. (2017). Offset-free multi-model economic model predictive control for changing economic criterion. *Journal of Process Control*.

[5] Ferramosca A., Limon D., Camacho E. F. (2014). Economic MPC for a changing economic criterion for linear systems. *IEEE Transactions on Automatic Control*.

[6] Geng Y., Wei Z., Zhang H., Maimaituerxun M. (2020). Analysis and prediction of the coupling coordination relationship between tourism and air environment: yangtze river economic zone in China as example. *Discrete Dynamics in Nature and Society*.

[7] Gordini N. (2014). A genetic algorithm approach for SMEs bankruptcy prediction: empirical evidence from Italy. *Expert Systems with Applications*.

[8] Jahedpari F., Rahwan T., Hashemi S. (2017). Online prediction via continuous artificial prediction markets. *IEEE Intelligent Systems*.

[9] Karanikić P., Mladenović I., Sokolov-Mladenović S., Alizamir M. (2019). Retraction Note: prediction of economic growth by extreme learning approach based on science and technology transfer. *Quality and Quantity*.

[10] Ataka K. (2014). Prediction of election result and economic indicator. *Resuscitation*.

[11] Lahmiri S. (2016). A variational mode decompoisition approach for analysis and forecasting of economic and financial time series. *Expert Systems with Applications*.

[12] Liu L., Wang Q., Wang J., Liu M (2016). A rolling grey model optimized by particle swarm optimization in economic prediction. *Computational Intelligence*.

[13] Nagy S., Pipek J. (2015). An economic prediction of the finer resolution level wavelet coefficients in electronic structure calculations. *Phys.chem.chem.phys*.

[14] Pipek J., Nagy S. (2013). An economic prediction of refinement coefficients in wavelet-based adaptive methods for electron structure calculations. *Journal of Computational Chemistry*.

[15] Rajsic P., Weersink A., Navabi A. (2016). Economics of genomic selection: the role of prediction accuracy and relative genotyping costs. *Euphytica*.

[16] Teljeur C., Neill M., Murphy L. (2014). Using prediction intervals from random-effects meta-analyses IN an economic model[j]. *International Journal of Technology Assessment in Health Care*.

[17] Vu H. L., Ng K. T. W., Bolingbroke D. (2019). Time-lagged effects of weekly climatic and socio-economic factors on ANN municipal yard waste prediction models. *Waste Management*.

[18] Yu W., Huafeng W. (2019). Neural network model for energy low carbon economy and financial risk based on PSO intelligent algorithms. *Journal of Intelligent and Fuzzy Systems*.

[19] Zhou L., Lai K. K., Yen J. (2014). Bankruptcy prediction using SVM models with a new approach to combine features selection and parameter optimisation. *International Journal of Systems Science*.

